# What’s Surprising About Surprisal

**DOI:** 10.1007/s42113-025-00237-9

**Published:** 2025-02-21

**Authors:** Sophie Slaats, Andrea E. Martin

**Affiliations:** 1 Max Planck Institute for Psycholinguistics, Wundtlaan 1, 6525 XD Nijmegen, The Netherlands; 2 Departement de Neurosciences Fondamentales, Université de Genève, Chemin Des Mines 9, 1202 Geneva, Switzerland; 3 Donders Institute, Radboud University, Kapittelweg 29, 6525 EN Nijmegen, The Netherlands

**Keywords:** Surprisal, Syntax, Language comprehension, Long short-term memory network

## Abstract

In the computational and experimental psycholinguistic literature, the mechanisms behind syntactic structure building (e.g., combining words into phrases and sentences) are the subject of considerable debate. Much experimental work has shown that surprisal is a good predictor of human behavioral and neural data. These findings have led some authors to model language comprehension in a purely probabilistic way. In this paper, we use simulation to exemplify why surprisal works so well to model human data and to illustrate why exclusive reliance on it can be problematic for the development of mechanistic theories of language comprehension, particularly those with emphasis on meaning composition. Rather than arguing for the importance of structural or probabilistic information to the exclusion or exhaustion of the other, we argue more emphasis should be placed on understanding how the brain leverages both types of information (viz., statistical and structured). We propose that probabilistic information is an important *cue* to the structure in the message, but is not a substitute for the structure itself—neither computationally, formally, nor conceptually. Surprisal and other probabilistic metrics must play a key role as theoretical objects in any explanatory mechanistic theory of language processing, but that role remains in the service of the brain’s goal of constructing structured meaning from sensory input.

**Supplementary Information:**

The online version contains supplementary material available at 10.1007/s42113-025-00237-9.

## Introduction

When we understand language, the task presented to the brain is to transform physical signals in the environment: from a continuous stream of speech or sign we perceive discrete words and combine them into phrases and sentences to form a structured and meaningful message. How exactly we perceive phrases and sentences from words (and morphemes) is the subject of considerable debate. A wealth of recent experimental work has shown that lexical probabilistic information is an incredibly good predictor of both behavior (e.g., reading times) and neural activity (e.g., EEG recordings) during language comprehension (e.g., Aurnhammer & Frank, 2019; Frank, 2013; Gillis et al., 2021; Monsalve et al., 2012; Weissbart et al., 2019). At first blush, this state of affairs seems to necessitate that probabilistic information plays a decisive role in comprehension, a conclusion drawn by many (among others, e.g., Armeni et al., 2019; Heilbron et al., 2022; Kuperberg & Jaeger, 2016). At the same time, there is ample evidence that human linguistic abilities go far beyond what even the largest probabilistic language models can do: we effortlessly interpret sentences that we never heard before—to wit, we understand just fine what was not likely; in fact, this is probably how we receive new information during conversation. As such, probabilistic information cannot be the whole story to language comprehension.

This paper is for all language researchers that want to understand how we understand and use, have used, or want to use surprisal and/or entropy in their models of language-related human data. In what follows, we will outline how the current focus on probabilistic information can sometimes be at cross purposes with uncovering the mechanisms that explain how we understand. Starting from central assumptions in theoretical linguistics, here, we use simulation to show that surprisal (and entropy) on its own (1) does not form a mechanistic account for the capacity we seek to explain and (2) cannot straightforwardly be compared to theory-driven predictors in analysis. Subsequently, we discuss that the modelling capacity of these metrics does not prove that language comprehension is fully subserved by prediction. Nevertheless, we know that both structure and statistics matter to the brain, both writ large and in the minutiae of language processing (e.g., Ding et al., 2016; Nelson et al., 2017b; Weissbart et al., 2019). Thus, rather than arguing for the importance of one to the exclusion or exhaustion of the other, we argue that more emphasis should be placed on how the brain leverages both to reach understanding and on how both shape processing. We propose that probabilistic information is an important *cue* to the structure in the message, but is not a substitute, functionally or otherwise, for the structure itself. Using surprisal and other probabilistic metrics as theoretical objects, and especially as *explanans*, lies at cross purposes with the goal of an explanatory and mechanistic theory of language comprehension.

## How to Describe a Sequence

Humans, like other organisms, strive to reduce uncertainty in their environment by learning, and by anticipating incoming sensory input (e.g., Friston, 2012; Hasson, 2017). In the case of quasi-sequential sensory input, which language is (both signed and spoken), there are multiple ways to learn about what was just perceived and to anticipate what comes next. One way to learn about our environment is by counting occurrences of the sensory events, remembering their sequential order, and tracking how often a given event follows another (e.g. Aslin & Newport, 2014; Linzen et al., 2017; Saffran et al., 1996). This is distributional information.

### Characteristics of Probabilities (an Introduction to Information Theory)

Distributional information for language comes in at least two flavors: *surprisal* and *entropy*. The two metrics are related, but differ in their predictive value, and have very distinct functional interpretations. Entropy is a measure of uncertainty about potential outcomes of future events, while surprisal is a post hoc measure of event expectancy. In language research, surprisal and entropy are typically calculated over sequences of words (see also: Hale, 2016).^1^ In practice, this means that a human or an algorithm keeps track of the frequency of occurrence of words in context.

*Surprisal* is calculated by taking the negative log-transformation of probability information of the word. In many cases, this is the conditional probability of the word: the probability of word given the *N* previous words. If a word is very likely to appear given the context, surprisal is low; it is high when the word does not often appear in the given context. In information theory, surprisal is also called *(Shannon) Information* (*I*) (Shannon, 1948). This is directly tied to the term “surprisal”: if a word is very likely to appear, the amount of information gained is low.^2^ Here, we will on occasion use *I* to denote surprisal. The equation of surprisal is shown in (1) below.

1 $$Surprisal\;I\left(w_i\vert w_{i-n}...w_{i-1}\right)=-\log\;\left(p\left(w_i\vert w_{i-n}...w_{i-1}\right)\right)$$

2 $$Entrophy\;H\left(w_i\vert w_{i-n}...w_{i-1}\right)=-\sum p\left(w_i\vert w_{i-n}...w_{i-1}\right)\ast\;\log\left(p\left(w_i\vert w_{i-n}...w_{i-1}\right)\right)$$

As is shown in (2) above, *entropy* is the weighted sum over the surprisal values of all the words that could appear in the position of the word in question. In other words, it is the *average surprisal* of all possible continuations; the expected amount of information for a continuation. Entropy depends both on the number of optional words, and on their probability distribution. If there are a lot of options, or if they all have the same probability of appearing, entropy will be high. On the other hand, if there are few options, or one of them has a much higher probability than the others (= lower surprisal!), entropy will be low. As such, entropy is a quantification of the uncertainty about what the transitional probability to the next word will be.

While the above examples concern probabilities of words, the equations are applicable to a wide variety of scenarios. In language, we can annotate a corpus for parts-of-speech and calculate surprisal of a noun given a verb and a determiner; or we can estimate the probabilities of letters and determine the entropy of the blank in “languag_”. Surprisal and entropy are distributional metrics in many studies, meaning that they are derived from the frequency of occurrence in a corpus. However, this is not always the case. It is also possible to set probabilities in a probabilistic context free grammar to reflect a priori preferences that are *not* derived from a text or speech database; see Hale (2016) for review. These probabilities give surprisal and entropy values too, but these do not find their origin in distributions. For this reason, in the remainder of this work, we will refer to surprisal values as being “probabilistic” rather than “distributional.”

Beyond language, we can apply the equations to anything sequential, such as observations of colors of cars: what’s the surprisal of a red car following two black ones given the cars we have observed on a given day, in a given location? What is the entropy of the event that follows “cooking” and “setting the table”? Given our knowledge of the likely sequence of events, entropy is low. Similarly, if “setting the table” were to be followed by “playing the recorder,” this event would have high surprisal. This may seem like a trivial observation, and it is, but it is an important one—more on this below—and it is easy to forget when we work with data and see how well metrics like lexical surprisal work for modeling and reconstructing human data.

### The Power of Surprisal

**Fig. 1:**
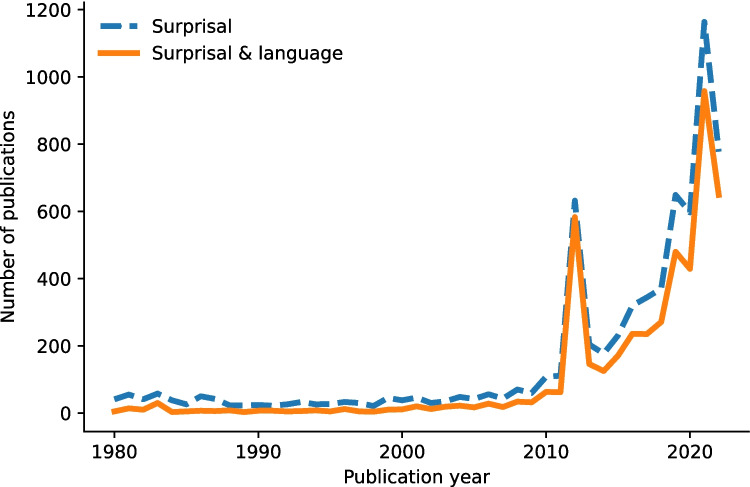
Number of publications between 1980 and 2022 that mention the keywords “Surprisal” or “Surprisal & Language,” obtained from Dimensions.ai on 15/05/2023

Probabilistic measures like (lexical) surprisal and entropy have been shown to be unequivocally robust predictors of brain activity and behavior. For example, higher surprisal values and a larger decrease in entropy both tend to lead to slower reading times (Aurnhammer & Frank, 2019; Frank, 2013; Hale, 2006; Levy & Gibson, 2013; Linzen & Jaeger, 2016; Pimentel et al., 2022; Smith & Levy, 2013). Corpus studies and computational models suggest that (backward) surprisal contains information about phrase structure (McCauley & Christiansen, 2019; Thompson & Newport, 2007). More recently, advances in neuroimaging and computational modeling alike have shown that oscillations in the delta, beta, and gamma bands show sensitivity to lexical surprisal (Gillis et al., 2021; Weissbart et al., 2019); that entropy reduction correlates with temporal lobe activity (Nelson et al., 2017a); and that surprisal and word frequency are tracked over and above acoustic and speech segmentation representations (Gillis et al., 2021). Furthermore, gamma power has been observed to increase when a word is highly predictable, but not to increase when it is not so predictable (Molinaro et al., 2013; Wang et al., 2012). In other words, surprisal and entropy are good predictors for behavioral and neurophysiological measurements, and the number of findings increases every day (see Fig. 1 below).

But the power of probabilistic information does not stop there. Some effects that are attributed to linguistic structure can be evoked by statistical regularities as well. In a seminal paper, Ding et al. (2016) showed that the occurrence rate of linguistic structures (syllables, phrases, and sentences) in speech are reflected in power in the neural signal at the corresponding frequencies. This effect was originally suggested to reflect the construction of linguistic units: the brain encodes abstract linguistic information. Nevertheless, since its publication several studies have shown that the low-frequency frequency tagging effects can be induced by transitional probability information alone (Bai, 2022; Batterink & Paller, 2017).

Evidence for the importance of probabilistic patterns in language (prior to the surge of large language models, e.g., Aslin & Newport, 2012; Elman, 1991; Monsalve et al., 2012; Saffran et al., 1996) has led to accounts that model (aspects of) language comprehension using probabilistic information, such as surprisal theory (Hale, 2001; Levy, 2008; Levy & Gibson, 2013) and entropy reduction theory (Hale, 2006). In these accounts, surprisal and/or entropy reduction are minimally taken to be an estimate of processing effort: comprehenders make use of probabilistic knowledge to predict both the structure of the input they have just heard or seen, and what they may encounter next. Processing difficulty varies according to the deviations from these predictions. Surprisal theory may not reject the notion of abstract syntactic structure (Hale, 2001, 2006; Levy, 2008): instead, it is, in essence, agnostic about the representations and mechanisms that lead to structure-dependent interpretation, or language comprehension. This is a logical consequence of surprisal, which can be calculated for any representation. Proponents of surprisal theory are explicit about this. E.g., Futrell, Gibson & Levy posit:

*“In addition to providing an intuitive information-theoretic and Bayesian view of language processing, surprisal theory has the theoretical advantage of being representation-agnostic: The surprisal of a word in its context gives the amount of work required to update a probability distribution over any latent structures that must be inferred from an utterance. [...] This representation-agnosticism is possible because the ultimate form of the processing cost function [...] depends only on the word and its context, and the latent representation literally does not enter into the equation.”* (Futrell et al., 2020, p. 4)

By consequence, authors often refrain from drawing conclusions about the computational, algorithmic or implementational levels of language comprehension, which are not the focus of surprisal theory (for a theory on how surprisal values come to reflect cognitive operations in comprehension, see Hale, 2014, Chapter 5). There are some extensions of surprisal theory that do include specifications of the cognitive architecture underlying comprehension. For example, Hale (2011) proposes that the next step in a parse is determined by the entropy of potential continuations at the current step, committing to a clear level of representation the metric is computed over, and to a theory of what this representation should be. In a different vein, Brouwer et al. (2021) provide a probabilistic instantiation of a “Retrieval-Integration Account,” a model that contains explicit levels of representation and two mechanisms: “retrieval,” the use of a word form and the context to access word meaning; and “integration,” the mapping of the word meaning and the prior context onto a representation of the utterance. Another proposal that goes beyond the notion of “modeling processing difficulty” is the work by Frank and colleagues. This work uses surprisal to focus on (the cognitive reality of) representational levels during language comprehension. Such studies question the necessity of abstract representations and (hierarchical) syntactic structure during language comprehension (Frank, 2013; Frank & Bod, 2011; Frank & Christiansen, 2018; Frank et al., 2012), but again do not offer an account of how surprisal values come to reflect comprehension nor of how particular meanings are perceived, but not others.

We highlight the representation-and-mechanism-agnosticism of probabilistic estimates, but our aim is to not criticize the literature around surprisal theory. Instead, we have two main objectives. Firstly, we wish to point language researchers that want to understand how we understand and use, have used, or want to use surprisal and/or entropy in their models of language-related human data to the issues that surround the use of metrics that are representation-agnostic, both from a psychological and from a cognitive neuroscientific perspective. More specifically, these issues arise because we are trying to explain the process of language comprehension, rather than describe it, with a model. Secondly, by focusing on syntactic representations, we will show that results obtained using surprisal or entropy, because they are representation-agnostic, do not warrant conclusions about the latent factors driving the surprisal estimates (as in Frank & Bod, 2011; Frank & Christiansen, 2018; Frank et al., 2012).

The goal is not to discourage the use of probabilistic estimates in our field—in our view they a core, crucial ingredient. In fact, in our models, probabilistic information derived from linguistic experience plays a role in shaping the process of language comprehension (e.g., Martin, 2016, 2020; Slaats et al., 2023, 2024). However, *despite* this role, the exclusive focus on representation-agnostic probabilistic information (no matter the level of representation or model used) may obscure the mechanism we need to explain how we understand.

### Characteristics of Structure: Explaining Compositional Meaning

One of the crucial mechanisms that our field seeks to explain is our capacity for *syntax*. Syntactic information is a description of the abstract collocation, constituency or dependency relations, and domains over which functions apply in human language. This information, these patterns, are the result of a system that can be described by rules: the grammar of a language. Decades of research in theoretical linguistics has provided insights into aspects of the grammar that are necessary to explain human competence in language production and comprehension. Firstly, central to the grammar is that the rules are structure-dependent rather than item-dependent: the rules apply to grammatical categories (“parts-of-speech”) and other rules, and not to the words themselves. Secondly, the structures generated by the grammar are *hierarchical*. This is a consequence of the observation that syntactic operations apply to *constituents*—(groups of) words that share a particular grammatical function—rather than to individual words (e.g., substitution: “yesterday I saw [my best friend]” – > “Yesterday I saw [her]”). In some cases, the same sequence of words can have more than one interpretation depending on the hierarchical relation between the elements; e.g. “[old men] and women” vs. “old [men and women]”; and “Robin saw [the woman with the binoculars]” vs. “Robin saw [the woman] with the binoculars.” These sequences are structurally ambiguous. Of course, only a subset of all possible word sequences is structurally ambiguous, but phrases are parsed hierarchically in all cases (Cinque, 2004; Coopmans et al., 2021; Everaert et al., 2015; Jackendoff, 1972).

Assuming the presence of syntax—an abstract, structure-dependent rule system that applies hierarchically—in language provides an explanation for the striking linguistic capacity to generalize, produce, and understand. Our knowledge of syntax is what allows us to produce and understand combinations of words that we have never perceived together, or to create sentences with novel words (e.g., Gertner et al., 2006). Most importantly, however, syntax is one of the elements that determine the meaning of a sentence, another being the meanings of the individual words (*principle of compositionality*). This capacity distinguishes language from other perception–action systems and makes language behavior difficult to account for (see Martin (2020) and Everaert et al. (2015) for discussion). The study of (a computational theory of) syntax has a long history in formal linguistics, but *how* this capacity is realized in mind and brain, at the algorithmic and implementational level, is an answer the field still aims to find—or *should* aim to find.

## Surprisal Is a Perfect Descriptor, but Not a Mechanism

Using simulation, we show that lexical surprisal values can reflect variance that finds its origin in the capacity we seek to explain (syntax), but despite this, these values themselves do not encode syntactic structure. Instead, surprisal likely contains information that derives from this structure. See the supplementary materials at https://osf.io/xp3r7/ and the code at https://github.com/sslaats/surprisal for the details of the used corpora and the model architectures. The statistical tests reported are two-tailed *t*-tests and Pearson’s correlation. The simulations described below serve an exemplifying purpose. Using a different model architecture will likely lead to numerically different results.

### Syntactic Structure Affects Surprisal Values

**Fig. 2:**
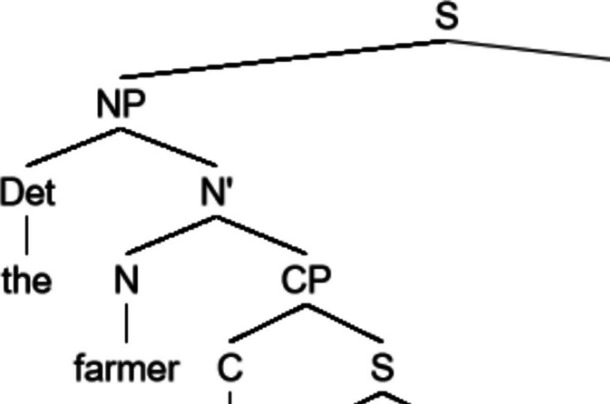
Example sentence generated using the toy grammar. The sentence reads: “the farmer that finds a toddler loves a scientist”

In the style of Elman (1991), we designed a phrase-structure grammar with 27 words and 4 parts of speech. We trained a long short-term memory model (LSTM) on the sentences generated with this grammar (see Fig. 2 for an example). To evaluate the effect of structure, a latent variable, on surprisal estimates, we trained a second LSTM on the same sentences, but with the words scrambled within each sentence. This method of scrambling maintains word frequency estimates, word frequency per sentence, and sentence length but removes all sentential structure. A comparison between the structured and scrambled models will provide insight into the impact of sentence structure on surprisal values. Both models were tested on sentences generated by the grammar that were not part of the training set.

**Fig. 3:**
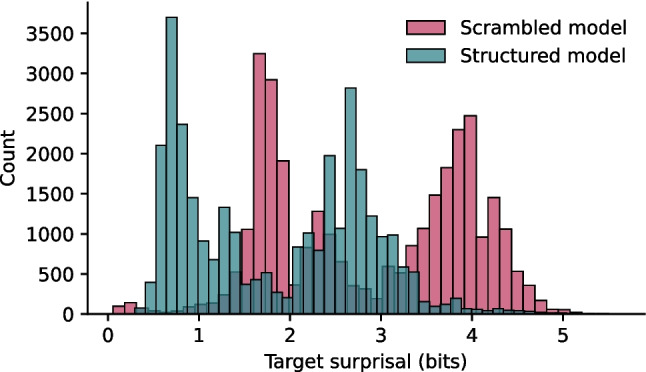
The presence of syntactic structure lowers the surprisal values of the words in sentences generated by a phrase structure grammar Surprisal values for each word in the test set for both structured (teal) and scrambled (pink) models

Compared to training on scrambled input, providing an LSTM model with structured input decreases surprisal values by 1.03 bit on average (*M*_struct_ = 1.86, sd_struct_ = 0.99; *M*_scram_ = 2.88, sd_scram_ = 1.08; *t*(62,854) = 124.08, *p* < 0.001; CI = [1.01, 1.04], Cohen’s *d* = 0.98, power = 1; Fig. 3). This illustrates that syntactic structure in the input impacts surprisal values. Nevertheless, the distributions of surprisal values for the structured and scrambled models are highly correlated (*ρ*(31426) = 0.92, *p* < 0.001), suggesting that the frequency of each individual word—constant between training corpora—does the heavy lifting when it comes to surprisal estimation.

**Fig. 4:**
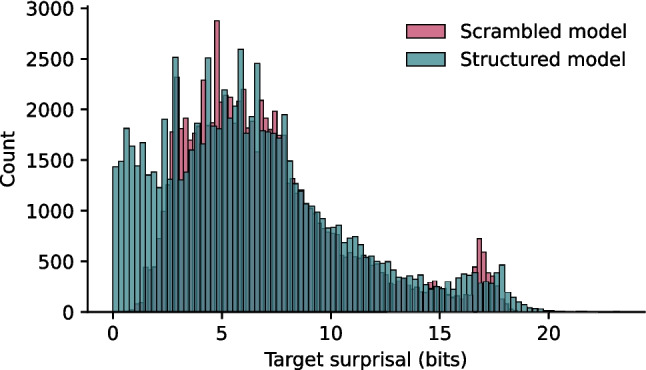
The presence of syntactic structure lowers the surprisal values of the words in sentences from the English OpenSubtitles 2018 corpus. Surprisal values for each word in the test set for both structured (teal) and scrambled (pink) models

Next, we tested whether these findings scaled to a larger corpus with a wider lexicon and a variety of sentence types. To this end, we repeated the same procedure with a larger model, trained on 700.000 words from an English corpus (OpenSubtitles 2018; Lison & Tiedemann, 2016). Though smaller, here, too we observe a difference between the distributions (*M*_struct_ = 6.42, sd_struct_ = 4.27; *M*_scram_ = 7.00, sd_scram_ = 3.68; *t*(160,916) = 29.18, *p* < 0.001; CI = [0.54, 0.62], Cohen’s *d* = 0.15, power = 1), with structured input decreasing the surprisal values by 0.58 bit on average (sd 1.82; Fig. 4). Also here, however, we observed a high correlation (*ρ*(80,457) = 0.91, *p* < 0.001) between the surprisal values estimated using the scrambled and structured model. In conclusion, syntactic structure affects the surprisal values of a large, naturalistic corpus in qualitatively similar ways to the effects observed in a small, constructed corpus. Thus, we show that (1) a decrease in surprisal is a general effect of the presence of language-like structure in sequences and that (2) a large part of the variance in surprisal values stems from unigram probability information.

### Surprisal Does Not Lead to Syntax

To show why surprisal cannot be a stand-in for theory, we ask an extreme question: can the underlying structure of a sequence (i.e., the latent syntactic structure that gives rise to the surface word order) be identified based on surprisal alone? To explore this, we trained and tested two additional LSTMs on the Spanish translation of the OpenSubtitles corpus (structured and scrambled), and we used a Random Forest Classifier to classify whether (groups of) surprisal values were coming from English or Spanish. This procedure was repeated on the scrambled variants of the models.

**Fig. 5:**
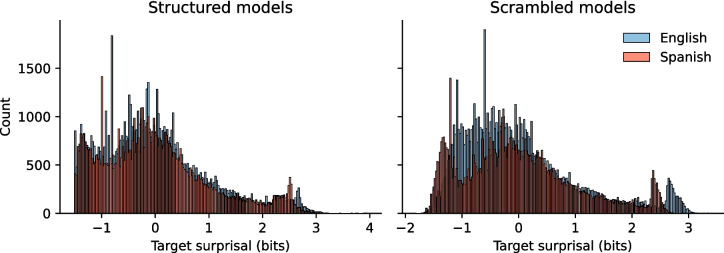
Z-scored surprisal values from the structured and scrambled models (English and Spanish). Observe the high peak in distribution in both languages; these are surprisal values for words that most often appear at the start of a sentence (Spanish: “no”; English: “I”)

The classifier performed above chance (structured: 63.8% (unigram) to 74.1% (10-gram)), indicating that surprisal values contain enough information for a classifier to distinguish between the two languages. However, this was also the case for the scrambled models (scrambled: 66.2% (unigram) to 84.2% (10-gram)), suggesting that structure is not the driving factor behind this above-chance performance. Instead, the classification appeared to be driven by uniqueness of surprisal values: each surprisal value was uniquely attributable to one or the other language. To remove this confound, we repeated the analysis with surprisal values rounded to the nearest 1 decimal. Doing so severely deteriorated the classification (structured 52.92% (unigram) to 67.98% (10-gram); scrambled: 58.46% (unigram) to 82.31% (10-gram)). In sum, (groups of) surprisal values contain enough information for a classifier to decide whether these values are coming from Spanish or from English, but this classification is crucially *not* dependent on structural properties of the language (see Fig. 5 below).^3^

Taken together, these two simulations paint the following picture. Lexical surprisal obtained from an LSTM encodes “structure” in the input, as is shown by a general decrease in surprisal. Nevertheless, structural differences are not the driving factor in the classification of patterns of surprisal values stemming from two structurally different languages. This shows that (patterns in) surprisal values can capture regularity in language in general, but do not encode language-specific aspects of structure.

### Understanding Does Not Depend on Surprisal

Consider the following Dutch sentences in (1). In each case, the verb *lag* (“lay”) is singular, hence asking for a singular subject to the sentence. The subject is different for every sentence: it is either semantically different (man or goldfish), or it is morpho-syntactically different (singular or plural). The subjects are printed in boldface.

**Table:** 

(1)	a.	Er	lag	een	**goudvis**	op straat	I = 15.4 bits
		There	lay	a	goldfish_[sin]_	in street	
		‘A goldfish_[sin]_ was lying in the street’					
	b.	Er	lag	een	**man**	op straat	I = 7.1 bits
		There	lay	a	man	in street	
		‘A man was lying in the street’					
	c.	*Er	lag	een	**goudvissen**	op straat	I = 21.9 bits
		There	lay	a	goldfish_[pl]_	in street	
		‘A goldfish_[pl]_ was lying in the street’					
	d.	*Er	lag	een	**mannen**	op straat	I = 14.9 bits
		There	lay	a	men	in street	
		‘A men was lying in the street’					

For every sentence, the surprisal value of the subject was calculated using a trigram model created with SRILM (Stolcke, 2002) trained on the Dutch OpenSubtitles 2018 corpus (Lison & Tiedemann, 2016). In other words, the surprisal value captures the surprisal of the subject in the context of the words “lag”, “een” – both requiring the next word to be a singular noun. The first observation is that the surprisal of the subject “goldfish_[sin]_” is higher than the surprisal of the subject “man”: 15.4 and 7.1 bits, respectively. This is expected: “man” is more frequent. If we change “man” to its plural “men”, or “goldfish” to its plural, the sentences become erroneous. In this case, surprisal value increases with approximately 7 bits, as well.

What is striking about this example is that the surprisal of the plural subject “men” is similar to that of “goldfish_[sin]_”—despite the plural subject rendering the sentences grammatically incorrect and, as such, uninterpretable without repair. Although we may not often encounter a goldfish on the street, this sentence is perfectly intelligible. This illustrates a crucial feature of language: understanding does not depend on surprisal (see also: van Schijndel & Linzen, 2021).^4^ More specifically, it shows that probability and grammaticality are theoretically distinct: the grammaticality of a sentence can change without affecting the surprisal values.

This highlights a crucial gap between probabilistic estimates such as surprisal and entropy and what *understanding* logically entails: reaching a single, interpretable representation of the input. Meaning can only arise when a stable representation has been formed. After all, probability distributions are not intelligible. Instead, the brain needs to converge on a discrete representation of the elements and the structure to reach understanding. Even if a large part of signal processing is probabilistic, at some point the brain has to “decide” or converge on a stable interpretation of what we are hearing, reading, or seeing. In fact, this is of one of the brain’s main features: it can take in probabilistic information and map it onto deterministic representations.^5^

To summarize, surprisal values are sensitive to structure in the input, but they do not uniquely capture the structure that generated the sequence, or grammatical well-formedness. These observations show why probabilistic metrics are not suitable as *explanans* for the core capacities of language.

## How Surprisal Can Obscure the View

Several studies that have used both predictors of syntactic structure and surprisal in their models have drawn conclusions similar to those outlined above: syntactic structure is necessary to create the best model of the data (Brennan et al., 2016; Brennan & Hale, 2019; Kapteijns & Hintz, 2021; Nelson et al., 2017b). Nevertheless, these studies report that probabilistic estimates, such as surprisal, explain more of the variance in the signal than do the syntactic predictors. The reason for this finding is that surprisal (even lexical) reflects variance from many latent factors. This is a consequence of the fact that surprisal estimates depend fully on the identity of the unit estimated. If we want to estimate the surprisal of a word, we need to know the identity of that word. This is the representation-agnostic character mentioned in the introduction: surprisal can—and will—parametrically reflect variation stemming from *any* domain or representational level of language, including syntax.

### Word Frequency and Word Order

**Fig. 6:**
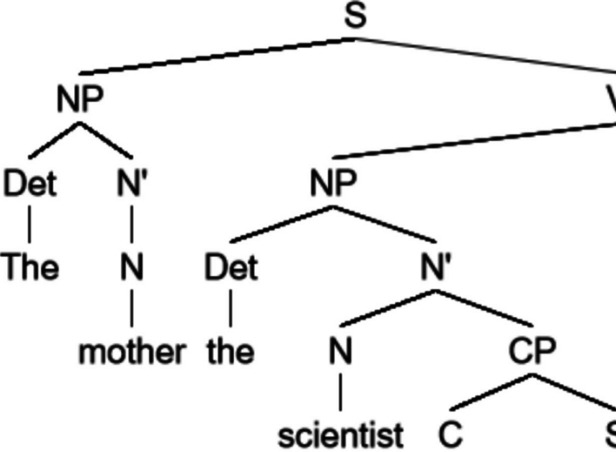
Example sentence generated using a small context-free grammar in which the constituent structure was changed to SOV. The sentence reads: “the mother the scientist that the bird discovers hears.” In the original subject-verb-object structure, that would be: “the mother hears the scientist that discovers the bird”

We demonstrate this by changing our toy grammar in two ways: by adapting the grammar itself, or by changing word frequency. To edit the grammar, we changed the order of the constituents in verb phrases. The complement (a noun phrase or a complementizer phrase) now precedes the verb. In other words, we have changed the grammar from “SVO” (subject-verb-object) to “SOV” (subject-object-verb); see Fig. 6 for an example sentence. Like in the scrambling models, doing so preserves the word frequency values as well as the number of words per sentence, but drastically changes the structure of the language. The model trained on this SOV-language was subsequently tested on the exact same test set as the previous models (structured and scrambled).

**Fig. 7:**
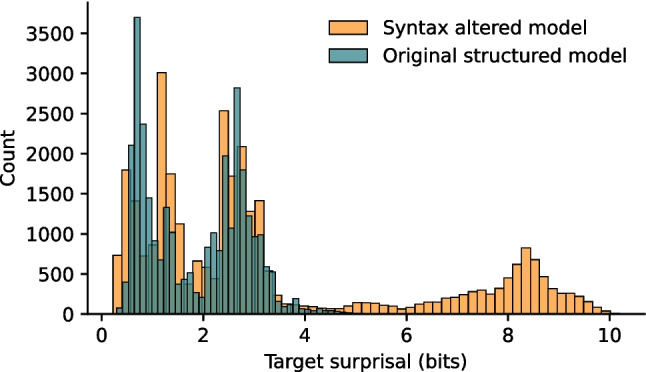
A different syntactic structure in the training input increases the surprisal values of the words in sentences generated by a phrase structure grammar. Surprisal values for each word in the test set for both SVO (teal) and SOV (yellow) models

Again, the resulting surprisal values from this SOV-trained model were significantly higher than those obtained using the original structured model^6^ (*M*_SVO_ = 1.86, sd_SVO_ = 0.99; *M*_SOV_ = 3.29, sd_SOV_ = 2.73; *t*(62,854) = 87.79, *p* < 0.001; CI = [1.41, 1.47], Cohen’s *d* = 0.7, power = 1) – unsurprising, because a large number of word-to-word transitions that were present in the test set were definitely *not* present in the training set by virtue of being ungrammatical (see Fig. 7).^7^ The correlation between the results from the structured model and the SOV-model was lower, but nevertheless still there (*ρ*(31,426) = 0.44, *p* < 0.001), indicating that unigram probability drives the pattern of surprisal values to an important extent. This is in line with observations from Hale (2016) and shown experimentally by Franzluebbers et al. (2024, p. 5).

**Fig. 8:**
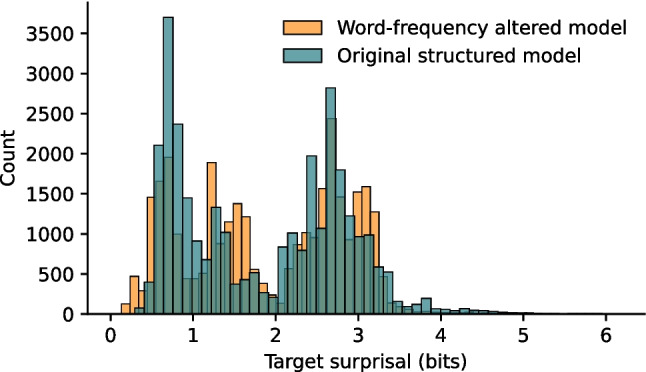
Surprisal values for each word in the test set from the corpora obtained with the original structured model and a model trained on a corpus that was adjusted for word frequency

Instead of changing the syntax, we can also change the word frequency values.^8^ To do this, the word frequency parameters were changed for a few words in the original SVO grammar. Specifically, the frequency of the words “woman,” “discovers,” and “a” was adjusted to be twice as high as the other words in their syntactic category.^9^ We then tested this model on the same test set again, and indeed: there was a significant difference between these distributions (*M*_SVO_ = 1.86, sd_SVO_ = 0.99; *M*_WF_ = 1.91, sd_WF_ = 0.97; *t*(62,854) = 6.78, *p* < 0.001; CI = [0.04, 0.07], Cohen’s *d* = 0.05, power = 1; see Fig. 8), while the correlation between the original and the word frequency adjusted estimates was still high (*ρ*(31,426) = 0.84; *p* < 0.001).

Indeed, both of these changes to the input/output relation—either the syntactic structure underlying the word sequences, or the frequency with which a word in a given category is selected—change the surprisal values we observe. Logically, beyond syntax, lexical surprisal values can reflect variability from all kinds of sources: lexical category, syntactic structure, the pragmatic and semantic context, priming, and so on (viz., any variable that affects word choice or word form). More than anything else, surprisal reflects language data—which is also what we provide to our participants by presenting stimuli. Surprisal values are calculated by passing the data through a probabilistic filter and thus are a prism or reflection of the data itself.^10^

Expressing the data through a probabilistic filter without being able to tease apart which variables in the data are contributing to a particular estimate of surprisal poses a problem when we want to use surprisal in combination with our readouts (e.g., behavioral and neural) to theorize about the cognitive architecture of the human language system. This is because while using a predictor that is derived from the data passed through a probabilistic filter may make it the most powerful predictor, it relegates the reasons why it is such a good predictor difficult to impossible to interpret: where do the effects come from? What linguistic factors create dynamics in surprisal? These observations should and do have important consequences for the way we develop our psycholinguistic theories, and for how surprisal comes into play in them. In short, surprisal does not allow us to draw conclusions about the potential mechanisms at work, e.g., in composition, even when its ability to predict the readout is robust.^11^

### Theory Is Always Wrong

Syntactic predictors in statistical models of reaction times or neural data are at a disadvantage even before running the model: the way they are constructed depends on a *theory of syntax* and of how this structure is implemented in the brain (Martin, 2020). Where surprisal values follow naturally from counting large numbers of words in a corpus,^12^ to create a syntactic predictor one must choose between numerous syntactic theories (for example, a minimalist or constructivist approach), make assumptions about the parsing strategy the brain employs to reach this structure (for example, “left-corner” or “top-down”; Brennan et al., 2016), and, finally, assume that the brain does not make any errors when parsing the input, for example when encountering ambiguous sentences (a so-called “perfect oracle”).

Any of these theory-driven assumptions will change the predictive power of the syntactic feature. This is not a problem in and of itself, but this becomes a problem when comparing it to a data-driven feature like surprisal. A data-driven estimate will perform better than a theory-driven estimate—because the data do not err, the theorizer does (Guest & Martin, 2021). These errors, or rectifications to those errors (when a theory-driven change to a feature affects the goodness of fit, or when an explicit experimental manipulation has a certain effect on responses), provide an opportunity to adjust our theory. By contrast, using surprisal as an explanation prevents us from looking at the influence from latent factors by reflecting variance that stems *from* these factors as a second-order variable. In this way, combined with representation-agnosticism, exclusive focus on surprisal as a predictor serves to obscure the mechanisms that explain behavioral and neural responses, and the view on the casual structures of human languages that shape its instantiation in the mind and brain.

## Prediction as a Mechanism

A mechanism that is sometimes invoked as the theory of how we understand language is *prediction*. In fact, surprisal is used as an operationalization of prediction (or even “strong prediction”: Michaelov et al., 2024). Predictive coding theories propose that our brain creates an internal model of the world which it constantly updates on the basis of the difference between the predicted input and the actual input (Clark, 2013; Feldman & Friston, 2010). Finding that surprisal models behavioral or neural data is taken by some as evidence that language comprehension hinges on predictive coding (Heilbron et al., 2022; Wacongne et al., 2011). Indeed, most operationalizations of surprisal are *forward* calculations, meaning that the surprisal value of the target word depends on the words before (as opposed to *backward* calculations, where surprisal is operationalized as the log probability of the target preceding the words that follow it). This gives the most common metric of surprisal a predictive component.

While we can appreciate that the appropriate use of predictive features can demonstrate that some anticipatory processes are happening in the brain or behavior during language comprehension, there are two problems. As we have seen in the paragraphs above, the use of surprisal as the predictive metric does *not prove* that the brain is generating predictions (solely) on the basis of language statistics (as suggested in Michaelov et al., 2024, among others). More importantly here, the existence of effects of predictive metrics do not provide evidence for prediction being necessary and sufficient for language comprehension. The reason is simple: predicting an item—or not predicting it, as in the case of high surprisal—does not entail understanding of the input. Imagine that you are visiting a country with a language you do not know. Every day, you pass by a mysterious sign with three words on it. You have no idea what it means, and yet, after spending some time in this place, when you see the first two words on the sign, you can predict the last one. During language comprehension, we can, and perhaps sometimes do (but probably not always, see Huettig & Mani, 2016), anticipate future linguistic content using our knowledge of language; the same knowledge that allows us to understand. In other words, if we are capable of understanding language, then we can anticipate some aspects of language in some circumstances; but the reverse does not hold.

## Pax Grammatica

As discussed in the “Characteristics of Structure: Explaining Compositional Meaning” section, the meaning of a linguistic expression is a function of the meaning of the individual words and the way they are combined. If the goal of the field is to understand how we understand, we must have a mechanistic account of this process, not only a predictive one. We need to understand what the relevant representations of the input are, how the brain represents them, and how the brain moves from one type of representation to another. To be more precise, we want to know (among other things), (if and) how our brain stores and accesses (quasi)lexical items, how these (quasi)lexical items are combined using knowledge of syntactic and semantic structure, (if and) how the representation of the “words” is separable from syntactic structure, and how the brain represents the “end product” (the meaning of the utterance).

**Fig. 9:**
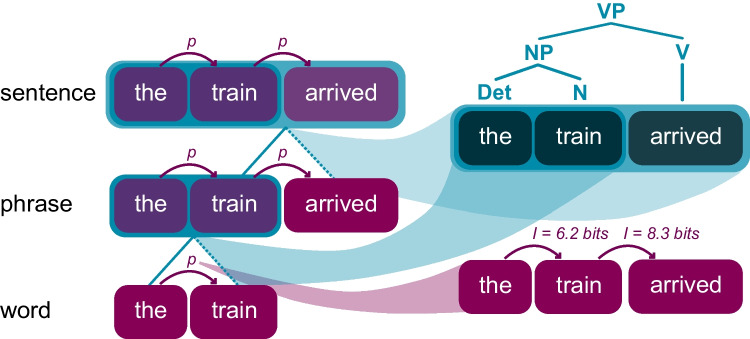
Schematic representation of the process of syntactic inference (left) and the differences between the outcome of this process (top right) and the outcome of lexical probabilistic information (bottom right). (The surprisal values are fictional.)

Surprisal, or the principles underlying the calculation of surprisal—alone—cannot serve as a mechanism for language comprehension, but the factors that give rise to surprisal effects likely play an outsized and valuable role in the building of a mechanistic theory of language comprehension. Consider Fig. 9 below. When the brain moves from representing the words in the bottom row to phrases and sentences in the rows above, *something* is done to the information. The probabilistic relation between the words ‘the, “train” and “arrived,” represented by the red arrows, may carry (non-deterministic) information about how the words are structurally related in the phrase or sentence—for example, if the surprisal of “arrived” is relatively high, this can signal the beginning of a new phrase (see Martin, 2016). But what *mechanism* is responsible for constructing the phrase (there are different proposals for this process, ranging from “construction” to “merge”), and (how) can this mechanism be implemented by the brain?^13^

Despite the arguments laid out in this paper, the notion that surprisal is not equivalent to structural information, composition, or comprehension, is not at all in conflict with the use of probabilistic information in sensory processing. In fact, that humans can make use of probabilistic information in their environment is undeniable: in the absence of any other sources of information such as meaning, prosody, or knowledge of linguistic structure, we are capable of using statistical information to segment the input stream, and, as such, “break in” to the realm of language (Aslin & Newport, 2012; Aslin et al., 1998; Batterink & Paller, 2019; Newport & Aslin, 2004; Saffran et al., 1996; Thompson & Newport, 2007; Trecca et al., 2019).

### Toy Model

In the process of comprehension, probabilistic information can play a similar role. Such an architecture could work as follows. For the purposes of this toy model, we assume a set of representational levels (not exhaustive by any means): phonetic, phonemic, syllabic, lexical, phrasal, sentential, and discourse-level. There is much evidence that linguistic levels of representation are separable in brain and behavior (e.g., Bai et al., 2022; Gwilliams et al., 2018; Kaufeld et al., 2020; Krauska & Lau, 2023; Leonard & Chang, 2014; Marslen-Wilson & Tyler, 2007; Mesgarani et al., 2014; Slaats et al., 2023; Ten Oever et al., 2022; Tezcan et al., 2023). For simplicity, we will focus only on how the brain performs *combination* (Pylkkänen, 2019) of units at a given level to infer the next level of representation. Within a given level of representation, then, we assume that the brain represents a sequence of units within this level of representation, in the spirit of either working memory, resonance (Jafarian & De Persis, 2015), attractors (Pascanu & Jaeger, 2011), or by retaining activation corresponding to a unit of representation (e.g., Gwilliams et al., 2018). In other words, previous input shapes the system in some way: either we retain several words prior to the processing current word, or the processing of previous words has changed the ongoing dynamics of the system such that the processing of the current word is shaped by these dynamics.

We propose that the brain represents probabilistic information *within* and *across* levels (viz., sequentially between phonemes, between words, between phrases, and bi-directionally between phonemes and words, words and phrases, etcetera) through *path dependence.* Path dependence means that the set of possible trajectories through state space is delimited by past trajectories (see Martin (2020) and Guest and Martin (2021)). By representing a sequence of units in this way, we gain access to transitional information, as well as information about how a specific unit relates to the level above; e.g., how likely it is for /*x*/ to be the start of a word. Extrapolating crucial effects from acquisition research (Aslin et al., 1998; Saffran, 2001; Thompson & Newport, 2007), we assume that we remain capable of using this probabilistic information to infer the underlying structure that gave rise to the sequence—a.k.a., to transform the information into the next level of representation (see Martin (2020) for pseudocode). This could mean the use of phonemic information to infer words (e.g., Tezcan et al., 2023), or words to infer phonemic information (e.g., Martin et al., 2017), or words to infer phrase structure or vice versa (e.g., Baese-Berk et al., 2019; Bai et al., 2022; Marslen-Wilson & Tyler, 1980; Thompson & Newport, 2007; van Alphen & McQueen, 2001).

What is crucial about this toy model is that probabilistic information is a factor, but it does not correspond to the representational levels themselves, nor to the computations that underlie the transformation of information that will eventually lead to a structured representation of the inferred meaning. Nevertheless, making use of probabilistic information as a *cue* to the level above (and, potentially, below; see Martin (2016) and Marslen-Wilson and Welsh (1978)) implies that these measures will affect the computations that do lead to the transformation of information, such as the inference of a phrase from words. Most of the findings in the domain of probabilistic information suggest effects of time, with most notably high surprisal being associated with longer reading times (Aurnhammer & Frank, 2019; Brothers & Kuperberg, 2021; Frank & Bod, 2011; Kapteijns & Hintz, 2021; Luke & Christianson, 2016; Monsalve et al., 2012), slower word recognition (Balling & Baayen, 2012), and so on. These findings suggest that probabilistic information may be a temporal modulator in the process of comprehension, affecting the time-course of the computations that lead to comprehension.

Rather than being the *mechanism* that leads to comprehension of what was said, we propose that lexical surprisal is a *cue* to detecting the presence and absence of phrase boundaries—much like how transitional probabilities were viewed by Saffran et al. (1996). Indeed, as mentioned in the “How to Describe a Sequence” section, (backward) surprisal contains information about phrase structure (McCauley & Christiansen, 2019; Thompson & Newport, 2007). This means that the surprisal of a given word can provide the recipient information about whether a phrase boundary is likely there, similar to prosody, co-articulation, and others (Martin, 2016, 2020),^14^ and is not an index of the process of composition itself. From a practical perspective, this suggests we should study interactions between (lexical) probabilities and syntactic operations rather than modelling surprisal and entropy as main effects—not only in behavior (e.g., Fine et al., 2013) but also in neuroimaging.

### Open Questions

For a human (brain) to be sensitive to a probabilistic estimate of any linguistic representation, this representation first needs to come into mental existence. What the nature of this representation is, and how it is inferred, are the difficult questions our field should aim to answer. The role of probabilistic cues is a part of the answer, and much about this aspect of the process is unknown. There is no consensus with respect to the nature of statistical information in the brain (how does the brain represent probability and/or uncertainty in general terms?), nor is it known what the brain “computes” statistical information over (which representations are probabilistic, and why?), and what mechanism is responsible for this “computation” (how does the brain keep track of probability?). In the field of language specifically, and cognitive science more generally, an important question is how the brain is capable to bootstrap structure (such as syntax) from statistics on the one hand. The reverse question is also open: does the brain refine probabilistic representations with structured knowledge, and if so, how does this work? We urge the field to consider these questions when using surprisal as a predictor for linguistic data.

## Conclusion

In this paper, we have shown that the current focus on probabilistic information in the psycholinguistic and neurolinguistic literature may prevent us from uncovering the mechanisms we need to explain how we understand. Using several simulations, we have illustrated that lexical and syntactic information are functionally inseparable, because such probabilistic information derives (in part) from syntactic information. This logically holds for any latent factor, not just for syntax. This means that any effects found for surprisal and entropy as derived from surface probabilities always leave room for the possibility of latent factors driving both the probabilities and the human responses, and do not allow any conclusions about which factors are involved (and why). Furthermore, being such a filter on many sources of linguistic information makes surprisal and entropy reliable predictors of human data. Because of this, these predictors will outperform predictors that represent the latent variables, the theoretical constructs we want to test and optimize. These issues make that surprisal and entropy are not, by themselves, suitable as *explanans* for the core capacities of language. Adopting surprisal and other probabilistic metrics as theoretical objects may distract us from the goal of an explanatory and mechanistic theory of language comprehension. We instead propose to view probabilistic information as a cue to the next level of abstraction; an aide to the mechanisms that we aim to uncover.

## Supplementary Information

Below is the link to the electronic supplementary material.

Supplementary file1 (DOCX 1013 KB)

## Data Availability

The code used for the simulations can be found in the following Github repository: https://github.com/sslaats/surprisal/. The generated corpora and model weights can be found on the Open Science Framework: https://osf.io/xp3r7/.

## Footnotes

1 Other measures of surprisal and entropy exist, but the lexical estimate plays a prominent role in recent research. For a note on other measures of surprisal and entropy, see the section 'Toy Model' below.

2 In psycholinguistics, this measure is often called *transitional probability* (TP). This term usually does not refer to surprisal obtained from neural network models.

3 As stated by Luce (2003), “the elements of choice in information theory are absolutely neutral and lack any internal structure; the probabilities are on a pure, unstructured set whose elements are functionally interchangeable.”

4 Values obtained from neural network models show the same pattern. Here, we use English sentences, and score them using the model from the English simulations described above (context of 10 words). Surprisal of **men* is lower than perfectly intelligible *kite*.

**Table:**

a.	On the street lies a **kite**	I = 14.46 bits
b.	On the street lies a **man**	I = 2.94 bits
c.	*On the street lies a **kites**	I = 16.45 bits
d.	*On the street lies a **men**	I = 7.88 bits

5 This statement is closely tied in with the question what mental representations of abstract concepts are like—and whether (large) language models “have” abstract representations. They do, in fact, not, as was already pointed out by Fodor and Pylyshyn (1988). For example, our original toy grammar chose a word with the correct part-of-speech 78% of the time. While this means that the model has learned something about the regularities of the language, this does *not* mean that the model has learned what a noun is. More sophisticated distributional representations, such as the internal states of neural networks., can be called “abstract” in the sense that a given pattern does not directly correspond to any individual item (e.g., when number of dimensions is reduced to below the number of types in the input). This does not mean that the model has an abstract representation, however. For example, two synonyms may have the same vector in a word2vec model, but that does not mean that the word2vec model understands “synonymy”; the model only represents that these words are likely to occur in the same contexts, statistically speaking (and this disregards the fact that some synonyms can be used in different contexts—such as different registers—, despite having the same referential meaning!). If the internal states of a sophisticated distributional model reveal a pattern that is consistent across all words that are synonyms of another word, then we could say that this model *statistically approaches* the concept “synonymy.” Nevertheless, this representation does not exist if we do not provide the model with a synonym. For humans, on the other hand, the concept “synonymy” persists in the absence of synonyms. The same holds for other subconscious knowledge of language, such as what a noun is, or what plurality means.

6 Notice that this SOV model is *also* structured.

7 This was crucially *not* the case in the scrambled model; any word-to-word transition was possible.

8 Obviously, word frequency itself is a distributional variable. This toy grammar does not have a semantics, pragmatics, or any other linguistic parameter that we can tweak; here, the word frequency parameter is causal for word frequency within a category. See the supplementary materials for details.

9 Essentially, this means that the lexical entropy in the training corpus is lower.

10 Devlin (2001, p. 21), cited in Luce (2003, p. 183): “Shannon’s theory does not deal with “information” as that word is generally understood. Instead, it deals with data-the raw material out of which information is obtained.”.

11 For a discussion of whether large neural networks can be a “mechanistic account” of cognition, see Guest & Martin (2023).

12 This may or may not be a simplification of what deep learning models are doing; see Carlini et al. (2021).

13 Obviously, the mechanism cannot be *prediction*. Firstly, we are perfectly capable of understanding sentences where some words have high surprisal (i.e., where the language model was wrong!); and secondly, and most importantly, predicting an item does not entail understanding of the input. See, e.g., Huettig & Mani (2016).

14 The status of *cue* rather than *mechanism* should hold at other levels of representation, too (e.g., syllables, phonemes, and phrases). Distributional information describes behavioral and neuroimaging data at all levels, ranging from the lower-level phonemic surprisal all the way to the higher-level syntactic estimates of surprisal (Brennan & Hale, 2019; Di Liberto et al., 2015; Heilbron et al., 2022). Being distributional estimates, the same problem holds: they prevent us from studying the underlying mechanism. For example, when recognizing words, the probabilistic relation between phonemes can provide information about where the input stream should be segmented into words—but it does not tell us how segmentation works at a mechanistic level, let alone how we recognize those segments as words.

## Code Availability

The code for this work is available at https://github.com/sslaats/surprisal.
