# Supplementary material for: What’s Surprising About Surprisal
Source: Comput Brain Behav. 2025 Feb 21;8(2):233–48. doi: 10.1007/s42113-025-00237-9 (PMC12125142; doi:10.1007/s42113-025-00237-9)
Supplement: Supplementary file 1 — Supplementary file1 (DOCX 1013 KB) [file 42113_2025_237_MOESM1_ESM.docx]

**Supplementary materials to Slaats & Martin (submitted)**

Methodological information for simulations. All code is available on <https://github.com/sslaats/surprisal>.

1. **Toy grammar-simulations**

The corpus was generated using a miniature phrase-structure grammar with four parts-of-speech: verbs (V), nouns (N), determiners (Det) and complementizers (Comp). The rules are displayed in (1).

1. Phrase-structure rules
   1. S --> NP VP
   2. CP --> Comp S
   3. NP --> Det N’
   4. N’ --> N
   5. N’ --> N CP
   6. VP --> V NP
   7. VP --> V CP

Using a small vocabulary of 27 words (see table 1 below), we generated a corpus of 10.000 sentences. The number of subordinate clauses was restricted to 5 irrespective of their binding position to avoid unrealistically long sentences and, more practically, an infinite loop.

Table 1. *Vocabulary used for the simulations*

| **Part-of-speech** | **Words** |
| --- | --- |
| Complementizer | *that* |
| Determiner | *a, the* |
| Noun | *woman, dog, goat, president, bird, colleague, mother, toddler, scientist, child, farmer, painter, cat* |
| Verb | *loves, discovers, reveals, notices, assumes, indicates, finds, senses, guarantees, teaches, hears, understands* |

Scripts used for these simulations:

- **grammar.py**: specifies the toy grammar (phrase-structure rules)
- **simulate-corpus.py**: uses the grammar to generate n sentences for training of the LSTM model
- **train-model.py**: train model on toy grammar
- **train-random-model.py**: train model on scrambled output of toy grammar
- **test-model.py**: test model trained on toy grammar
- **compare-models.py**: compares the surprisal values on the test set between scrambled and structured models
- **language.csv**: the vocabulary & POS to use for simulate-corpus.py

The model weights for these simulations are in the subfolder ‘model-weights’; the training corpora are in the folder ‘corpora’. Surprisal values for the test sets are in the folder ‘results’. All on <https://osf.io/xp3r7/>.

- 1. **Simulation: ‘syntax leads to surprisal’**
     1. **Model training & testing**

We split the corpus into a training- and testing set with a ratio of 80/20, and used the training set to train a recurrent neural network model with an embedding layer of 10 nodes, a hidden LSTM layer of 64 nodes, and a linear layer mapping back to the word space. The learning rate was 0.1 and we used negative log likelihood loss as implemented in PyTorch (Paszke et al., 2019).

This yielded a ‘structured model’; the input to the LSTM model was generated by a grammar. We also created a ‘scrambled model’. To create the scrambled training set, we randomly shuffled the words within each sentence from the training set. This method preserves word frequency across the corpus, individual sentence length, and the number of words from a certain part-of-speech in each sentence, but removed all structure. We then trained an LSTM model with the same architecture as the structured model to obtain the scrambled model. For both the structured and the scrambled model, the input required a 10-word context, meaning that we extracted 10grams for every sentence prior to training.

We estimated surprisal values for every word in the test set using the scrambled and the structured model. The test set was identical in both cases (the output from the grammar).

- - 1. **Results**

In the histogram above in Figure 1, one can clearly see that providing the LSTM model with structured input (the blue bars) decreases surprisal values by 1.06 bit on average (t = 127.08, p < 0.001). This clearly shows that surprisal values can reflect syntactic structure. Nevertheless, the correlation between the surprisal values from the scrambled and structured models is 0.92 (p < 0.001).


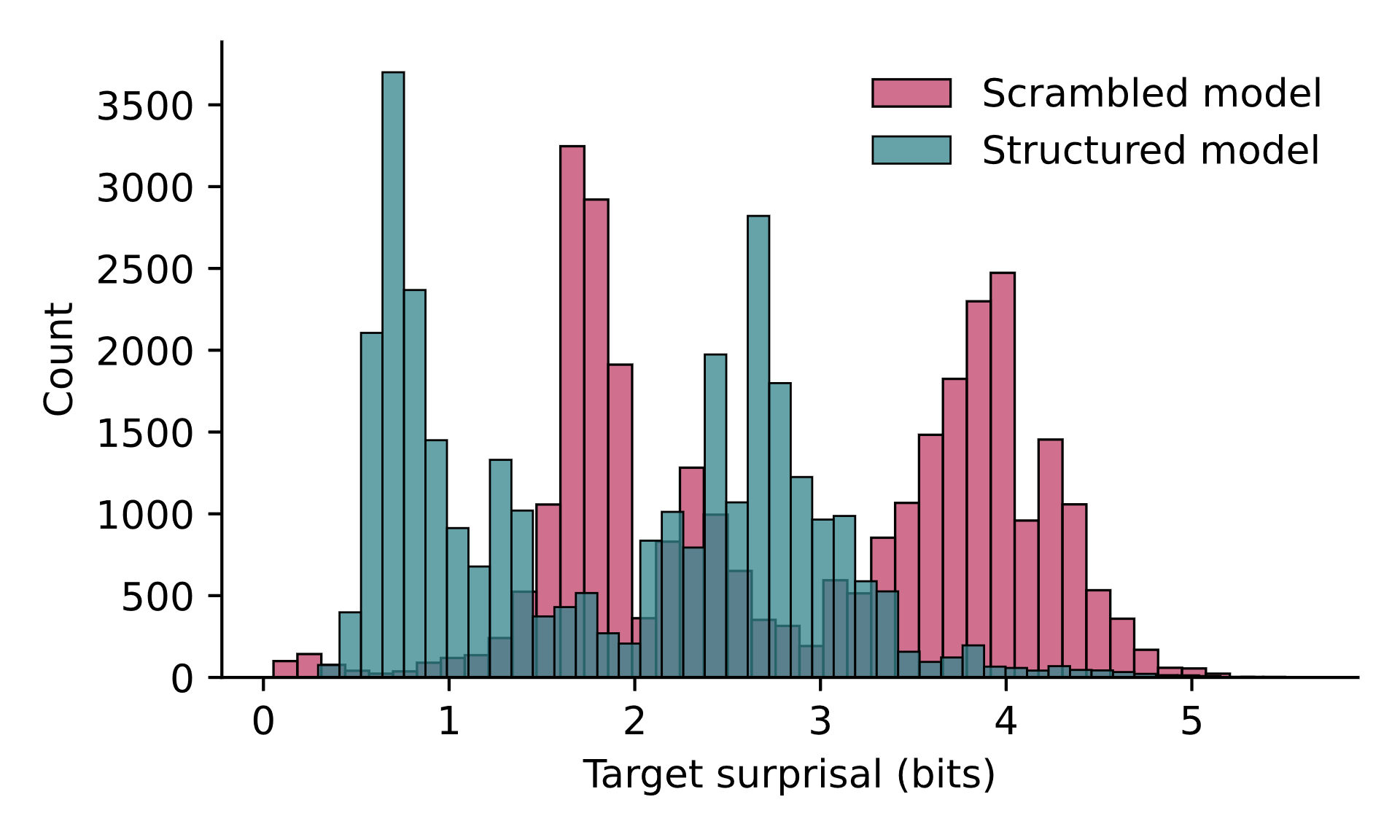


Figure 1. *Surprisal values for each word in the test set from the corpus obtained with a phrase-structure grammar.*

- - 1. **Model predictions**

The random model predicts the correct word approximately 16% of the time. The model defaults to predicting determiners with the occasional complementizer; these are the most frequent words in the corpus, and will therefore most often be correct. These two categories make up 45% of the total corpus, and there are three options: ‘the’, ‘a’, and ‘that’. Out of these 45%, the network is correct at chance; 1/3^rd^ of the time. This yields 15% correct – so the model essentially performs at chance. The same is the case for the accuracy in part-of-speech; there are four options, and the model predicts the correct part-of-speech 27% of the time. For the structured model we see a slightly different pattern. The model predicts the correct word in approximately 28% of the cases. This model also defaults to a small set of words (noun = ‘scientist’, sometimes ‘child’; verb = ‘finds’), but these words match the correct part-of-speech 78% of the time; most of the failures are in the complement of the VP or NP, where a complementizer or a determiner are both good continuations of the sentence.

- 1. **Simulation: ‘surprisal obscures the view’, syntax**

To edit the grammar, we changed the order of the constituents in verb phrases. The complement (a noun phrase or a complementizer phrase) now precedes the verb. In other words, we have changed the grammar from “SVO” (subject-verb-object) to “SOV” (subject-object-verb); see figure 6 for an example sentence. Doing so preserves the word frequency values as well as the number of words per sentence, but drastically changes the structure of the language.

- - 1. **Model training and testing**

The model parameters were the same as the previous simulation. The model trained on this SOV-language was subsequently tested on the exact same test set as the previous models (structured and scrambled).

- - 1. **Results**

The resulting surprisal values were significantly higher than those obtained using the original structured model^[[1]](#footnote-1)^ (t=87.79, p < 0.001) – unsurprising, because a large number of word-to-word transitions that were present in the test set were definitely *not* present in the training set because they were **ruled out** by the grammar^[[2]](#footnote-2)^. The correlation between the results from the structured model and the SOV-model was lower, but nevertheless still there (ρ = 0.44, p < 0.001). In other words, a difference between the syntax of the input to the model and the sentences or words the model is tested on, will lead to higher surprisal values.

- - 1. **Model predictions**

Lexical accuracy was 20.4%, lower than the original model; also the POS accuracy was lower than the original model (58.9%).

- 1. **Simulation: ‘surprisal obscures the view’, word frequency**

The word frequency parameters were adjusted for a few words in the original SVO grammar. Specifically, we adjusted the frequency of the words ‘woman’, ‘discovers’, and ‘a’ to be twice as high as the other words in their syntactic category (nouns, verbs, and determiners, respectively). Essentially, this means that the lexical entropy in the training corpus is lower.

- - 1. **Model training and testing**

The model parameters were the same as the previous simulation. The model trained on this WF-adjusted-language was subsequently tested on the exact same test set as the previous models (structured and scrambled).

- - 1. **Results**

We then tested this model on the same test set again, and indeed: there was a significant difference between these distributions (t = 6.78, p < 0.001), while the correlation between the original- and the word frequency adjusted estimates was still high (ρ = 0.84; p < 0.001).


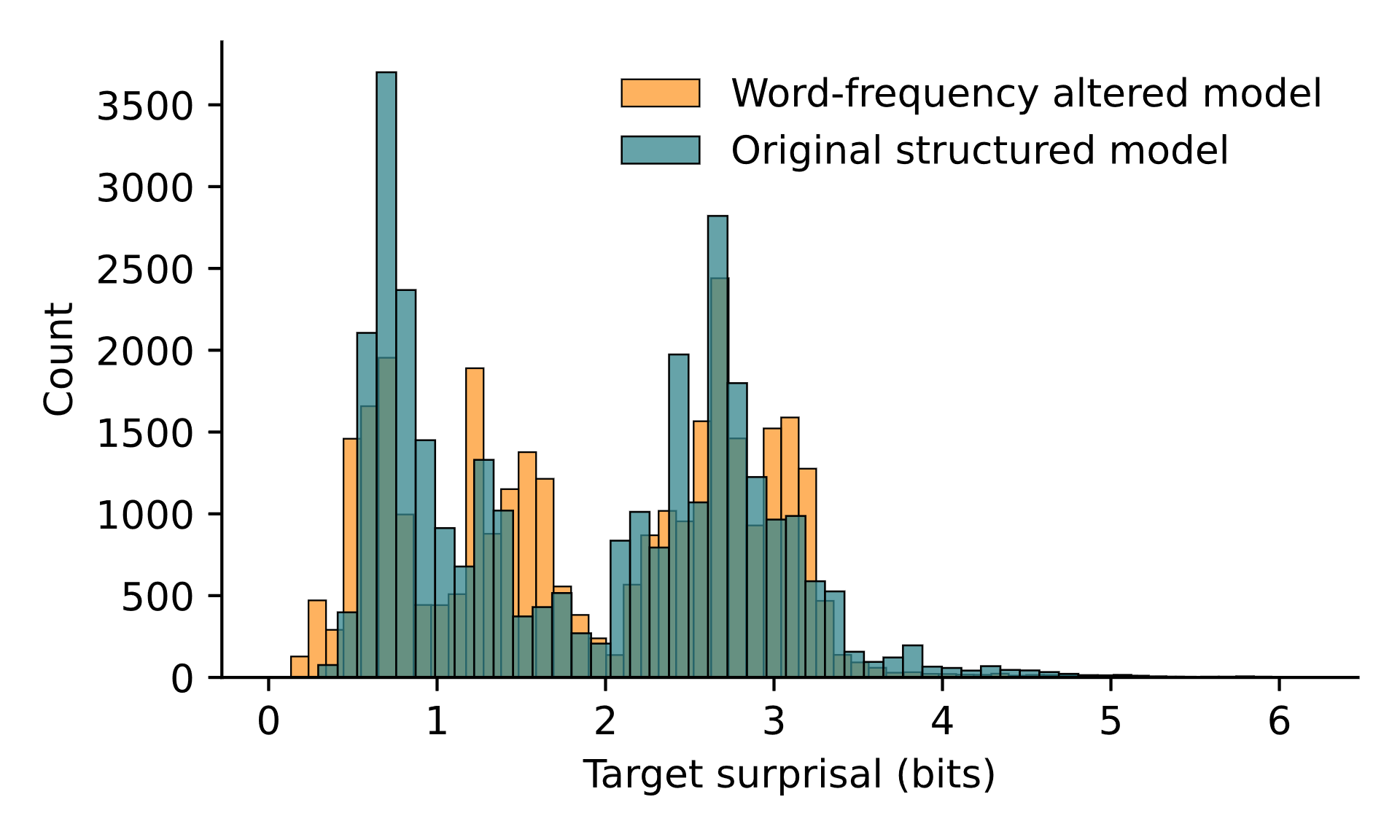


Figure 2. *Surprisal values for each word in the test set from the corpora obtained with the original structured model and a model trained on a corpus that was adjusted for word frequency.*

Lexical accuracy was slightly lower than the original (27.9%); POS accuracy was te same (76.3%).

1. **Natural language**

The corpora used for these simulations were obtained from the OpenSubtitles project (Lison & Tiedemann, 2016).

Scripts used for these simulations:

- **preprocessing-opensubtitles.py**: sentence & word tokenization and interpunction removal of OpenSubtitles corpus
- **train-model-natural-1layer.py**: train model on OpenSubtitles corpus
- **test-model-natural.py**: test model trained on OpenSubtitles corpus
- **correlation-natural.py**: compares the surprisal values on the test set between scrambled and structured models
- **clustering.py**: use a RandomForestClassifier to classify surprisal values as coming from Spanish or English

The weights for these models can be made available upon request. The corpora can be downloaded from <https://opus.nlpl.eu/OpenSubtitles-v2018.php>. Surprisal values for the test sentences can be found in the folder ‘results’.

- 1. **Simulation: ‘syntax leads to surprisal’, part II**
     1. **Model training & testing**

We trained a recurrent neural network with a 300-node embedding layer, a 600-node LSTM-layer, and a linear layer on approximately 118.000 English sentences (roughly 800.000 words) to predict the next word using a context of 10 words on a sentence-by-sentence basis. and a linear layer mapping back to the word space. The learning rate was 0.1 and we used negative log likelihood loss as implemented in PyTorch (Paszke et al., 2019).

Like before, we trained two models: a structured model, trained on intact sentences from the corpus; and a scrambled model, trained on sentences in which the word order was randomized. This method of scrambling maintains word frequency estimates, word frequency per sentence, and sentence length, but removes all sentential structure. We tested both models on the same test set of 10.000 sentences (approximately 70.000 words).

- - 1. **Results**

While the difference between the random and the structured models is much smaller, here too we observe a difference between the distributions (t = 29.18, p < 0.001); the mean difference in surprisal values is 0.58 bit (sd 1.82). Here too, however, we observed a correlation of 0.91 (p < 0.001) between the surprisal values estimated from the scrambled and structured model.


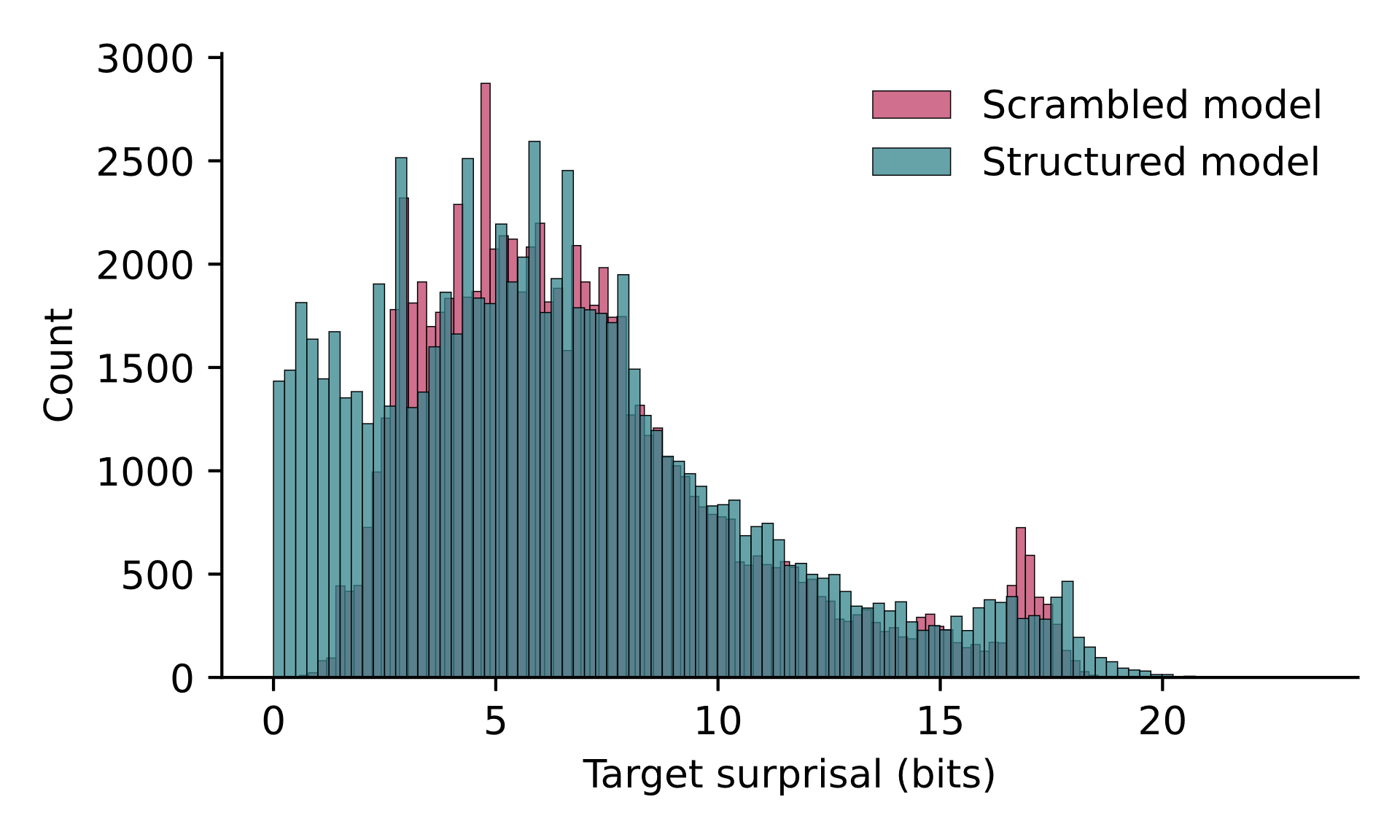


Figure 3. *Surprisal values for each word in the test set from the OpenSubtitles 2018 corpus obtained with a phrase-structure grammar.*

- 1. **Simulation: ‘surprisal does not lead to syntax’**

For this simulation, we trained two additional models on the Spanish translation of the English corpus. This was a corpus of approximately 116.000 sentences (roughly 800.000 words).

- - 1. **Model training & testing**

The training and testing procedures were identical to those of the English model described in 2.1.1 above.

- - 1. **Classification**

Before classification, we z-scored the surprisal values to account for the possibility that one of the languages is generally more surprising than the other. Subsequently, we trained a Random Forest Classifier (100 estimators as implemented in Scikit-Learn (Buitinck et al., 2013)) on 80% of these sentences to predict whether the surprisal values belonged to English or to Spanish. Since structure may be encoded in patterns of surprisal values rather than the individual values, we did this for a range of groups of surprisal values (from unigrams to 10-grams). The distribution of the surprisal values is visible in figure 4.


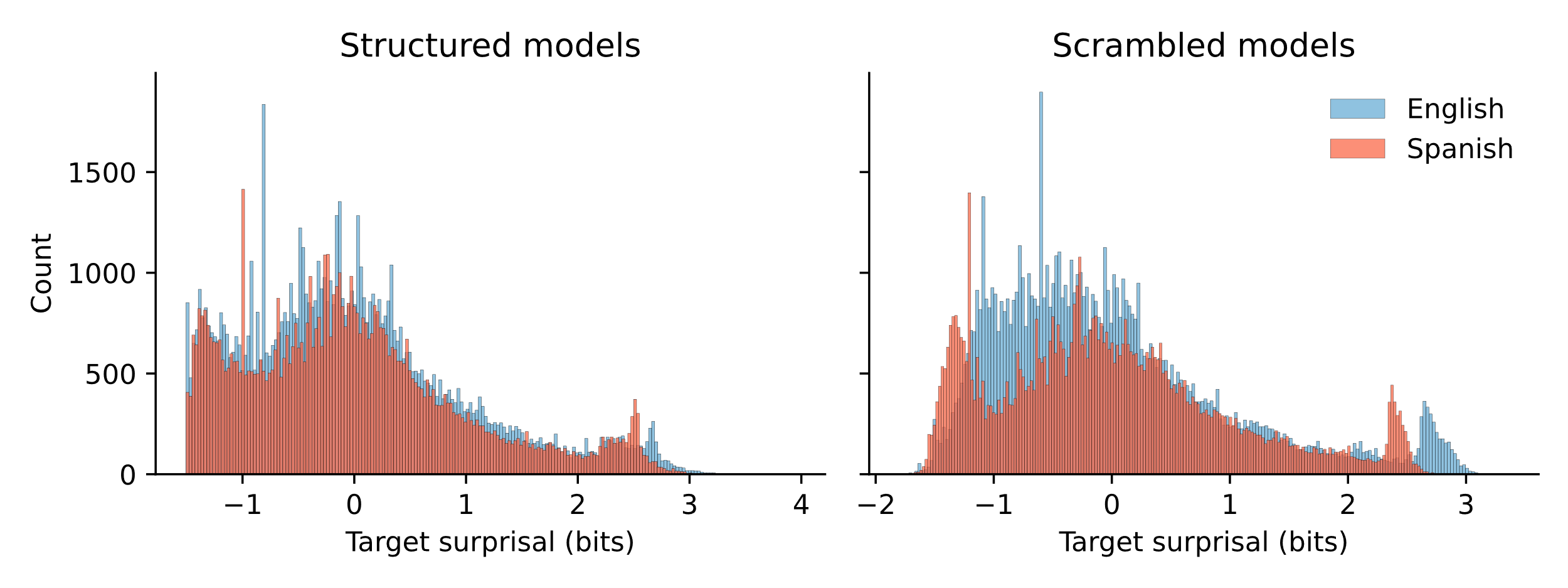
Figure 4. *Z-scored surprisal values from the structured- and scrambled models (English and Spanish). Observe the high peak in distribution in both languages; these are surprisal values for words that most often appear at the start of a sentence (Spanish: ‘no’; English: ‘I’).*

- 1. **Results**

We found that the classifier was able to predict with 63.8% accuracy if a single surprisal value belonged to the English or the Spanish grammar, and this increased to 74.1% for groups of 10 surprisal values With chance at 50% and 10.000 testing items, this means that the classifier performs above chance. We could have stopped here, and concluded that we were wrong: surprisal values *do* map back onto structure. But alas, we did not. We trained the same classifier on the results from the Spanish and English *scrambled* models (the words shuffled within each sentence; see the distribution in figure 4). Despite these models not containing *any* structural information, the classifier performed at 66.2% for unigram surprisal values, and performance increased to 84.2% for 10 surprisal values. Apparently, surprisal values from the scrambled model are easier to attribute to one or the other language than those from the structured models.

Why do these classifiers work at all? Structure is not the driving factor, apparently. No, specific surprisal decimal values appeared to be one of the driving factors. The surprisal values were uniquely attributable to one or the other language due to high specificity of the values. In other words, each surprisal value was unique to either Spanish or English, and the classifier learned this (partially). We tested if the pattern in groups of surprisal values was strong enough for the classifier to attribute the values to either language by rounding all values to 1 decimal. This preserves a potential structure-specific pattern, but removes the uniqueness. This change decreased the classifier’s accuracy in both the structured- and the scrambled model (structured: accuracy ranges from 52.92% (unigram) to 67.98% (10-gram); scrambled: accuracy ranges from 58.46% (unigram) to 82.31% (10-gram)), but the accuracy values were still higher for the scrambled model than for the structured model.

**References**

Buitinck, L., Louppe, G., Blondel, M., Pedregosa, F., Mueller, A., Grisel, O., Niculae, V., Prettenhofer, P., Gramfort, A., Grobler, J., Layton, R., VanderPlas, J., Joly, A., Holt, B., & Varoquaux, G. (2013). API design for machine learning software: Experiences from the scikit-learn project. *ECML PKDD Workshop: Languages for Data Mining and Machine Learning*, 108–122.

Lison, P., & Tiedemann, J. (2016). *OpenSubtitles2016: Extracting Large Parallel Corpora from Movie and TV Subtitles*.

Paszke, A., Gross, S., Massa, F., Lerer, A., Bradbury, J., Chanan, G., Killeen, T., Lin, Z., Gimelshein, N., Antiga, L., Desmaison, A., Kopf, A., Yang, E., DeVito, Z., Raison, M., Tejani, A., Chilamkurthy, S., Steiner, B., Fang, L., … Chintala, S. (2019). PyTorch: An Imperative Style, High-Performance Deep Learning Library. In H. Wallach, H. Larochelle, A. Beygelzimer, F. d’Alché-Buc, E. Fox, & R. Garnett (Eds.), *Advances in Neural Information Processing Systems* (Vol. 32). Curran Associates, Inc. https://proceedings.neurips.cc/paper/2019/file/bdbca288fee7f92f2bfa9f7012727740-Paper.pdf

1. Notice that this SOV model is *also* structured. [↑](#footnote-ref-1)
2. This was crucially *not* the case in the scrambled model; any word-to-word transition was possible. [↑](#footnote-ref-2)
